# Association between triglyceride glucose-body mass index and MASH, cardiovascular disease in MASLD patients: a cross-sectional study and machine learning analysis

**DOI:** 10.1016/j.metop.2025.100423

**Published:** 2025-11-19

**Authors:** Xiaoxuan Tang, Fenglan Wang, Yiran Liu, Yujia Gao, Mengxi Li, Chong Liu, Duanming Zhuang, Bin Zhang

**Affiliations:** a Department of Gastroenterology, Nanjing Drum Tower Hospital, Affiliated Hospital of Medical School, Nanjing University, Nanjing, China; b Department of Gastroenterology, Gaochun People's Hospital, Nanjing, China

**Keywords:** Triglyceride glucose-body mass index, Metabolic dysfunction-associated steatotic liver disease, Metabolic dysfunction-associated steatohepatitis, Cardiovascular disease, Machine learning

## Abstract

**Background:**

The triglyceride glucose-body mass index (TyG-BMI) is a useful marker for metabolic dysfunction-associated steatotic liver disease (MASLD) and cardiovascular disease (CVD). However, its ability to differentiate metabolic dysfunction-associated steatohepatitis (MASH) and CVD risk among patients with MASLD requires further investigation. This study evaluates the association between TyG-BMI and MASH/CVD risk in patients with MASLD and applies machine learning (ML) to identify relevant risk factors.

**Methods:**

The study used data from National Health and Nutrition Examination Survey (NHANES) to analyzed associations between TyG-BMI and two clinical outcomes: MASH, defined by the FibroScan-AST (FAST) score ≥0.35, and self-reported CVD. Participants were categorized based on TyG-BMI quartiles: Q1 (<246.79), Q2 (246.79–280.32), Q3 (280.33–323.99), and Q4 (≥324.00). Multivariate survey-weighted logistic regression and restricted cubic splines (RCS) were used to assess relationships and potential nonlinearity. Multiple ML models were employed, with feature importance interpreted via Shapley Additive Explanations (SHAP) analysis.

**Results:**

Among 674 MASLD participants (390 males), higher TyG-BMI was independently associated with increased risks of MASH and CVD risk. Compared with Q1, Q4 had adjusted odds ratios of 24.46 (95 % CI: 2.94–203.31, P = 0.003) for MASH and 3.53 (95 % CI: 1.26–9.90, P = 0.017) for CVD. RCS indicated linear relationships between TyG-BMI and both outcomes. The gradient boosting machine and support vector machine exhibited the optimal performance best in discriminating high-risk MASH (ROC: 0.910) and CVD (ROC: 0.773), confirming TyG-BMI as a significant risk factors.

**Conclusion:**

TyG-BMI effectively identifies MASH and CVD risk in patients with MASLD, offering clinicians a practical tool for risk stratification and management.

## Abbreviations

Adaboost: Adaptive boosting
ALT: Alanine aminotransferase
AST: Aspartate aminotransferase
AUROC: Area under the receiver operating characteristic curve
CAP: Controlled attenuation parameter
Catboost: Categorical features gradient boosting
CHD: Coronary heart disease
CHF: Congestive heart failure
CVD: Cardiovascular disease
DCA: Decision curve analysis
DNL: De novo lipogenesis
EPV: Events-Per-Variable
FAST: FibroScan-AST
FFA: Free fatty acid
GBM: Gradient boosting machine
HbA1c: Glycosylated hemoglobin
HCC: Hepatocellular carcinoma
HDL-C: High density lipoprotein cholesterol
IR: Insulin resistance
IQR: Interquartile range
KNN: K-nearest neighbors
LDL-C: Low density lipoprotein cholesterol
LR: Logistic regression
LSM: Liver stiffness measurement
MASLD: Metabolic dysfunction-associated steatotic liver disease
MASH: Metabolic dysfunction-associated steatohepatitis
NAFLD: Non-alcoholic fatty liver disease
NASH: Non-alcoholic steatohepatitis
NHANES: National Health and Nutrition Examination Survey
PA: Physical activity
RF: Random forest
ROS: Reactive oxygen species
SHAP: Shapley Additive Explanations
SMOTE: Synthetic Minority Over-sampling Technique (SMOTE)
STROBE: Strengthening the Reporting of Observational Studies in Epidemiology
SVM: Support vector machine
T2DM: Type 2 diabetes mellitus
TG: Triglycerides
TyG-BMI: Triglycerides glucose-body mass index
XGBoost: Extreme gradient boosting

## 1 Introduction

Metabolic dysfunction-associated steatotic liver disease (MASLD) is the most prevalent chronic liver disease worldwide, and its progressive form, metabolic dysfunction-associated steatohepatitis (MASH), can lead to hepatic fibrosis, cirrhosis, and hepatocellular carcinoma [1,2]. The identification of reliable, non-invasive biomarkers is crucial for risk stratification and timely intervention in MASLD, as highlighted by recent seminal studies in the field [[3], [4], [5]]. Building upon this foundation, our study evaluates the composite triglyceride glucose-body mass index (TyG-BMI). Insulin resistance (IR) and obesity represent key pathological drivers of MASH(6), highlighting the need for biomarkers that accurately capture these metabolic dysfunctions. The TyG-BMI, a composite index integrating triglycerides, glucose, and body mass index, has emerged as a promising, cost-effective surrogate marker for IR and obesity-related metabolic risk [[7], [8], [9], [10]].

Cardiovascular disease (CVD) stands as the leading cause of mortality in the MASLD population [11]. Research indicates that the risk of CVD in patients with MASLD is significantly higher than in those with simple fatty liver or metabolic dysfunction [12]. Moreover, synergism exists between MASLD and traditional cardiometabolic risk factors, (including hypertension, diabetes, and hyperlipidemia), further enhancing the risk of cardiovascular events [13].

IR is one of the fundamental pathological mechanisms underlying of MASLD, and TyG index serves as a non-invasive alternative measure of IR. IR reduces liver glucose utilization by the liver and promotes fatty acid synthesis and storage, leading to steatosis [6]. In addition, it inhibits fat breakdown, reduces fat mobilization, and aggravates liver fat deposition in the liver [14]. The TyG index showed strong correlation with triglyceride levels, a key contributor to hepatic steatosis. This association promotes both production and release of free fatty acids (FFAs), thereby exacerbating fat accumulation in the liver. [15]. The TyG index correlates with inflammation and oxidative stress, which in turn activates NF-κB and other pathways, leading to hepatic inflammation and fibrosis, and hepatocellular injury [16]. A significant positive association exists between increase in BMI levels and impaired IR [17]. Obesity is also associated with increased oxidative stress, which further damages liver cells and promotes the development of liver steatosis [18]. A recent Chinese study has demonstrated that TyG-BMI can effectively predict the progression of non-alcoholic steatohepatitis (NASH) and liver fibrosis in patients with non-alcoholic fatty liver disease (NAFLD) [19]. However, the discriminatory ability of TyG-BMI for identifying MASH and CVD in the American population, as well as within the context of newly proposed MASLD remains unclear. Therefore, this study aimed to investigate the association between TyG-BMI and the risks of MASH and CVD in the United States MASLD population, as recently defined.

## 2 Methods

### 2.1 Study population

**Fig. 1:**
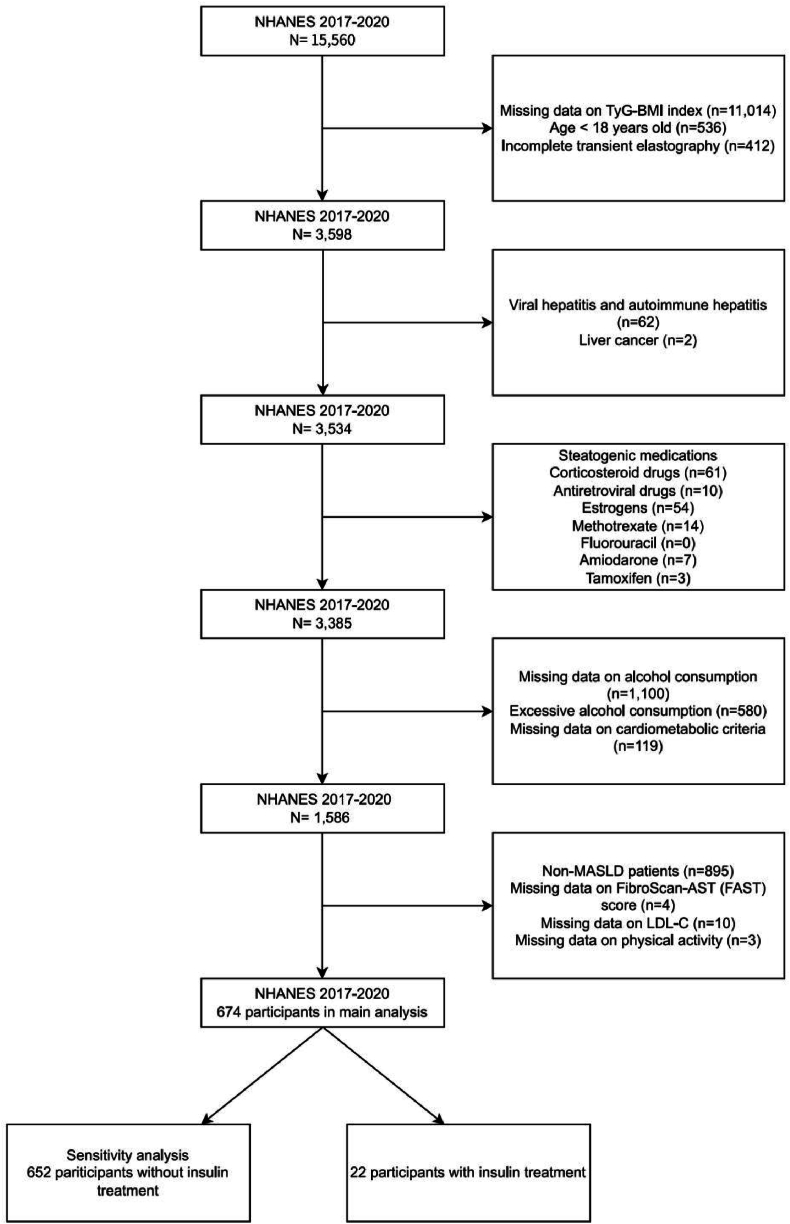
Flow chart of participants included in this study.

This study used data obtained from the U.S. National Health and Nutrition Examination Survey (NHANES) 2017–2020 cycle. Researchers implemented a sophisticated stratified multistage probability sampling design to ensure nationally representative population coverage across key demographic variables including age, sex, race, and socioeconomic status, thereby enhancing the external validity of the study findings. The initial cohort comprised 15,560 participants. A stepwise exclusion process was applied: First, individuals with missing TyG-BMI index data (n = 11,014), age <18 years (n = 536), or incomplete transient elastography results (n = 412) were excluded, leaving 3598 participants. Next, individuals with viral hepatitis, autoimmune hepatitis (n = 62), or liver cancer (n = 2) were excluded, resulting in 3534 participants. Other exclusions criteria were individuals who use steatogenic medications users (e.g., corticosteroid drugs, antiretroviral drugs; total n = 149), leaving 3385 participants. Subsequently, individuals with missing alcohol consumption data (n = 1100), excessive alcohol intake (n = 580), or incomplete cardiometabolic criteria (n = 119) were excluded, leaving 1586 participants. Finally, non-MASLD patients (n = 895), those missing FibroScan-AST (FAST) scores (n = 4), low density lipoprotein cholesterol (LDL-C) data (n = 10), or physical activity records (n = 3) were excluded, resulting in a final cohort of 674 patients with MASLD for the primary analysis. For sensitivity analysis, the cohort was stratified into non-insulin-treated (n = 652) and insulin-treated (n = 22) subgroups. The selection of the study population is shown in Fig. 1. All NHANES participants provided written informed consent prior to data collection, ensuring compliance with ethical standards and lawful, transparent data utilization. The current investigation strictly followed STROBE (Strengthening the Reporting of Observational Studies in Epidemiology) guidelines for observational epidemiological research to maintain methodological integrity and reporting transparency.

### 2.2 Measurement and calculation of TyG-BMI index

The TyG-BMI index combines both IR through TyG index and adiposity (through BMI), serving as a comprehensive marker of metabolic dysfunction. This composite index offers clinical advantages of being easily obtainable and cost-effective, as it requires only three routinely measured parameters: fasting triglycerides (TG), glucose, and BMI. The TyG index was calculated using the logarithmic transformation of fasting glucose and triglyceride values according to the established formula: ln[fasting glucose (mg/dL) × fasting triglycerides (mg/dL)/2] [20]. BMI was calculated as weight in kilograms divided by height in meters squared. The composite index TyG-BMI index was subsequently calculated by multiplying these two derived values. This calculation method followed established protocols validated in previous research.

The study population was stratified into four groups of equal size according to the quartile distribution of the TyG-BMI index distribution: Q1: TyG-BMI <246.79 (n = 168); Q2: 246.79 ≤ TyG-BMI <280.32 (n = 168); Q3: 280.33 ≤ TyG-BMI <324.00 (n = 168); Q4: TyG-BMI ≥324.00 (n = 170).

### 2.3 Definition of high-risk MASH

According to the 2023 international consensus criteria [1], MASLD was diagnosed based on the following three mandatory components: First, presence of hepatic steatosis assessed via vibration-controlled transient elastography (FibroScan®) with a controlled attenuation parameter (CAP) threshold of ≥269 dB/m [21]. Second, secondary causes of steatosis were excluded, including: excessive alcohol intake (>3 standard alcoholic drinks/day for men, >2 standard alcoholic drinks/day for women); viral hepatitis; autoimmune hepatitis or other chronic liver diseases. Third, presence of at least 1 cardiometabolic criteria.

The diagnosis of high-risk MASH was based on a FAST score (FibroScan-AST score)≥0.35 [22,23]: The FAST score, a non-invasive index combining liver stiffness measurement (LSM), CAP and aspartate aminotransferase (AST), indicates high likelihood of MASH (sensitivity ≥80 %, specificity ≥90 %). To further enhance the robustness of our study, we additionally used a FAST score of ≥0.67 to define high-risk MASH. This approach balanced accuracy and feasibility for population-based studies, avoiding the need for invasive liver biopsy.

### 2.4 Definition of CVD

CVD was identified based on self-reported physician diagnoses obtained from the NHANES medical conditions questionnaire. Specifically, participants were asked: “Has a doctor ever told you that you have congestive heart failure (CHF), coronary heart disease (CHD), angina, heart attack, or stroke?”. An affirmative response to any of these conditions was classified as the presence of CVD.

### 2.5 Covariates: definition and data collection

The following covariates were included to adjust for potential confounders [1]: demographic characteristics: age, gender, race [2]; life style factors: smoking status(judged by smoking questionnaires), physical activity (PA) (classified by physical activity questionnaire criteria) [3,24]metabolic and clinical measures: hypertension (blood pressure≥140/90 mmHg or antihypertensive medication use), diabetes (defined by any of the following criteria: fasting glucose ≥7.0 mmol/L, glycosylated hemoglobin (HbA1c) ≥6.5 %, or a self-reported history of physician-diagnosed diabetes.), AST, alanine aminotransferase (ALT), HbA1c, high density lipoprotein cholesterol (HDL-C), LDL-C [4]; use of medications such as statins and insulin.

### 2.6 Statistical analysis

The subjects were divided into groups Q1 to Q4 based on the quartiles of the TyG-BMI index. Mean ± standard deviation was used to present normally distributed continuous variables, and *t*-test was used for groups comparisons; median (interquartile range, IQR) was used to present non-normally distributed continuous variables, and Wilcoxon-test was used for groups comparisons. Categorical variables were represented by frequency (percentage), and groups comparisons were performed using Pearson's chi-squared test or Fisher's exact test. Two logistic regression models were established to evaluate high-risk MASH and CVD according to the TyG-BMI index: Model 1 (unadjusted model): no covariates were adjusted, and independent effect of the TyG-BMI index was assessed. Model 2 (fully adjusted model): adjusted for the following confounding factors: age, gender, race, smoking status, physical activity, hypertension, diabetes, AST, ALT, HbA1c, HDL-C, LDL-C, use of statins, use of insulin, with group Q1 as the reference. This study used restricted cubic splines (RCS) to explore the nonlinear relationship between the TyG-BMI index and the outcome events. To further enhance the robustness of the study, the analysis was repeated after excluding all participants receiving insulin treatment. The subgroup analyses were performed to verify robustness.

The analysis was completed using R software (version 4.2.0) and STATA (version 16.0), with NHANES complex sampling weights, and defined P < 0.05 as statistically significant on both sides.

### 2.7 Machine learning model development and performance evaluation

To ensure the robustness of our machine learning models and adhere to the Events-Per-Variable (EPV) guideline, we employed the Boruta algorithm and lasso regression to identify key discriminatory variables, followed by random allocation of patients into training (70 %) and validation (30 %) sets. Synthetic Minority Over-sampling Technique (SMOTE) was used to address class imbalance. Complex survey weights were not incorporated as they are not compatible with the SMOTE resampling procedure. Cross-validation was used to robustly select the optimal parameters and mitigate overfitting during the hyperparameter tuning phase. Ten machine learning models were developed using the selected features, and categorized into three groups: traditional models, ensemble learning methods, and neural networks. The traditional models included logistic regression (LR) for its interpretability in modeling linear relationships, support vector machines (SVM) effective for small-sample high-dimensional data, and k-nearest neighbors (kNN) as a similarity-based nonparametric approach [25,26]. Ensemble learning comprised random forests (RF), recognized for noise resistance in high-dimensional data, gradient boosting machines (GBM), extreme gradient boosting (XGBoost) and Light GBM as optimized boosting frameworks supporting parallel computation and automatic handling of missing values, along with adaptive boosting (AdaBoost) for iterative weight adjustment and categorical features gradient boosting (CatBoost) excelling in categorical feature processing. Neural networks were implemented to capture complex nonlinear patterns [27,28]. Model performance was primarily assessed using the area under the receiver operating characteristic curve (AUROC), supplemented by decision curve analysis (DCA) to quantify clinical net benefit. The best-performing model on the validation set was selected as the best model, which then underwent Shapley Additive Explanations (SHAP) analysis to quantify variable contributions through importance rankings and dependence plots.

## 3 Results

### 3.1 Baseline characteristics of participants

As shown in Table 1, the baseline characteristics analysis of 674 MASLD patients stratified by TyG-BMI index quartiles revealed a significant metabolic deterioration across increasing TyG-BMI groups (Q1-Q4). Patients in Q4 group (TyG-BMI≥324.00) were younger than those in Q1 group (median 46 vs 57 years, P = 0.002) but had markedly higher diabetes prevalence (27.85 % vs 10.46 %, P = 0.005). Participants in Q4 group exhibited higher liver stiffness (indicated by LSM median 6.1 kPa vs 4.6 kPa in Q1) and steatosis severity (indicated by CAP 343.65 dB/m vs 293 dB/m), both P < 0.001, than did those in the Q1 group. The incidence of high-risk MASH increased to 18.61 % in the Q4 group vs. 2.26 % in the Q1 group (P < 0.001), whereas CVD prevalence showed no significant difference (8.68 % vs 7.94 %, P = 0.604). Moreover, the Q4 group had higher ALT (24 U/L vs 19 U/L, P = 0.001) and HbA1c (5.7 % vs 5.4 %, P = 0.001), but lower HDL-C (41 mg/dL vs 59 mg/dL, P < 0.001). Notably, no statistical differences across groups were observed with respect to sex distribution, race distribution, smoking status, physical activity, hypertension prevalence, AST, LDL-C, insulin use and statin use showed no statistical differences across groups. The baseline characteristics of patients stratified by FAST score categories are detailed in Supplementary Table 1. Compared to the low-likelihood group (FAST <0.35, n = 606), both the indeterminate-risk (FAST 0.35–0.66, n = 51) and high-risk MASH (FAST ≥0.67, n = 17) groups had a significantly lower proportion of females, a higher prevalence of hypertension, and higher levels of AST, ALT and TyG-BMI index (all P < 0.05).

**Table 1:** Baseline characteristics of patients grouped according to the TyG-BMI index quartiles (n = 674).

Characteristics	Q1 <246.79(n = 168)	Q2 246.79–280.32(n = 168)	Q3 280.33–324.00(n = 168)	Q4 >324.00(n = 170)	P value
Demographic Characteristics					
Age(years)	57.000 [45.000, 68.000]	54.000 [40.116, 65.433]	54.850 [39.000, 63.000]	46.000 [33.000, 60.000]	0.002
Gender,n(%)					0.281
Man	105(57.56 %)	104(67.98 %)	101(58.36 %)	80(52.75 %)	
Woman	63(42.44 %)	64(32.02 %)	67(41.64 %)	90(47.25 %)	
Race,n(%)					0.111
Mexican American	18(6.09 %)	27(11.27 %)	31(10.38 %)	25(11.82 %)	
Other Hispanic	14(5.98 %)	17(4.91 %)	15(5.74 %)	22(9.60 %)	
Non-Hispanic White	71(73.45 %)	69(69.76 %)	65(69.17 %)	56(60.06 %)	
Non-Hispanic Black	24(5.12 %)	26(6.72 %)	36(7.01 %)	52(13.90 %)	
Other Race	41(9.36 %)	29(7.34 %)	21(7.70 %)	15(4.61 %)	
Lifestyle Factors					
Smoker,n(%)					0.635
No	101(42.56 %)	90(44.27 %)	96(36.66 %)	107(37.33 %)	
Yes	67(57.44 %)	78(55.73 %)	72(63.34 %)	63(62.67 %)	
PA,n(%)					0.242
No	100(59.91 %)	100(61.67 %)	97(60.03 %)	91(48.74 %)	
Yes	68(40.09 %)	68(38.33 %)	71(39.97 %)	79(51.26 %)	
Disease Status					
Hypertension,n(%)					0.174
No	98(58.82 %)	77(54.26 %)	67(43.01 %)	72(46.91 %)	
Yes	70(41.18 %)	91(45.74 %)	101(56.99 %)	98(53.09 %)	
Diabetes,n(%)					0.005
No	140(89.54 %)	124(82.52 %)	111(75.78 %)	103(72.15 %)	
Yes	28(10.46 %)	44(17.48 %)	57(24.22 %)	67(27.85 %)	
Laboratory Parameters					
AST(U/L)	19.000 [17.000, 22.000]	20.000 [16.000, 24.000]	21.000 [17.000, 24.999]	20.000 [17.000, 25.000]	0.314
ALT(U/L)	19.000 [14.000, 24.000]	21.000 [16.000, 28.303]	24.000 [19.000, 33.159]	24.000 [17.000, 37.000]	0.001
HbA1c(%)	5.400 [5.200, 5.734]	5.500 [5.200, 5.800]	5.600 [5.328, 6.000]	5.700 [5.500, 6.100]	0.001
HDL-C(mg/dL)	59.466 [41.000, 71.260]	48.000 [41.000, 56.000]	44.000 [39.000, 54.000]	41.000 [37.000, 48.000]	<0.001
LDL-C(mg/dL)	114.745 [88.391, 138.000]	113.000 [89.000, 133.000]	113.000 [81.788, 136.500]	115.000 [92.000, 130.000]	0.921
Treatment					
Statin,n(%)					0.416
No	121(71.75 %)	113(72.59 %)	112(65.59 %)	134(78.03 %)	
Yes	47(28.25 %)	55(27.41 %)	56(34.41 %)	35(21.97 %)	
Insulin,n(%)					0.442
No	166(99.23 %)	159(97.47 %)	160(97.37 %)	167(98.63 %)	
Yes	2(0.77 %)	9(2.53 %)	8(2.63 %)	3(1.37 %)	
Outcomes					
High-risk MASH,n(%)					<0.001
No	163(97.74 %)	158(96.61 %)	147(88.84 %)	138(81.39 %)	
Yes	5(2.26 %)	10(3.39 %)	21(11.16 %)	32(18.61 %)	
CVD,n(%)					0.604
No	159(92.06 %)	148(87.45 %)	144(87.28 %)	153(91.32 %)	
Yes	9(7.94 %)	20(12.55 %)	24(12.72 %)	17(8.68 %)	
LSM(kPa)	4.600 [4.000, 5.500]	4.674 [4.000, 5.600]	5.400 [4.400, 6.700]	6.100 [4.800, 8.215]	<0.001
CAP(dB/m)	293.000 [278.441, 307.120]	295.980 [284.222, 326.000]	308.000 [291.248, 333.000]	343.648 [313.000, 374.750]	<0.001

Values are weighted.Bold indicates P value < 0.05. MASLD, metabolic dysfunction associated steatotic liver disease; PA, physical activity – meeting MET (≥600 MET-minutes/week, equivalent to 150 min/week of moderate-intensity or 75 min/week of vigorous-intensity physical activity); MET: metabolic equivalent minutes of moderate to vigorous physical activity per week; AST, aspartate aminotransferase; ALT, alanine aminotransferase; HbA1c, glycated hemoglobin; HDL-C, high density lipoprotein cholesterol; LDL-C, low density lipoprotein cholesterol; High risk MASH, high risk metabolic dysfunction-associated steatohepatitis–FAST score ≥0.35; FAST, FibroScan-AST; CVD, cardiovascular disease; LSM, liver stiffness measurement; CAP, controlled attenuation parameter.

### 3.2 Associations between the TyG-BMI index and high-risk MASH in participants with MASLD

Logistic regression analyses demonstrated significant associations between TyG-BMI index quartiles and high-risk MASH (Table 2). In Model 1 (unadjusted), participants in the Q3 group (odds ratio (OR) = 5.42, 95 %confidence interval (CI):1.47–19.99) and Q4 group (OR = 9.87, 95 %CI:2.79–34.92) showed markedly elevated MASH risk compared with that for those in the Q1 group (reference), whereas Q2 group showed no significant association (OR = 1.52, 95 %CI:0.41–5.66). In Model 2 (adjusted for age, gender, race, smoking status, physical activity, hypertension, diabetes, AST, ALT, HbA1c, HDL-C, LDL-C, statin use, and insulin use), the Q3 and Q4 groups showed significant association with higher incidence of high-risk MASH (Q3: OR = 6.99, 95 % CI: 1.23–39.78; Q4: OR = 24.46, 95 % CI: 2.94–203.31).

**Table 2:** Associations between TyG-BMI index and high-risk MASH^a^ (n = 674), NHANES 2017–2020.

	TyG-BMI index			
	Q1	Q2	Q3	Q4
Model 1				
OR (95 %CI) P-value	1.00	1.52(0.41,5.66) 0.535	5.42(1.47,19.99) 0.011	9.87(2.79,34.92) <0.001
Model 2				
OR (95 %)CI P-value	1.00	1.71(0.27,10.65) 0.566	6.99(1.23,39.78) 0.028	24.46(2.94,203.31) 0.003
Sensitivity analysis after exclusion of participants receiving insulin treatment (n = 652)				
Model 1				
OR (95 %CI) P-value	1.00	1.08(0.28,4.15) 0.914	5.22(1.39,19.57) 0.014	9.96(2.81,35.26) <0.001
Model 2				
OR (95 %)CI P-value	1.00	0.84(0.11,6.44) 0.867	4.48(0.84,23.94) 0.079	18.22(2.52,131.90) 0.004

Model 1: Non-adjusted model.

Model 2: Adjusted for:age; gender; race; smoker; PA; hypertension; diabetes; AST; ALT; HbA1c; HDL-C; LDL-C; statin; insulin.

a High risk MASH was dignosised by FAST score ≥0.35.

To enhance the robustness of the study, sensitivity analysis excluded patients receiving insulin treatment (n = 652). In Model 2, the Q3 and Q4 groups were still retained robust associations with the outcome (Q3: adjusted OR = 4.48, P = 0.079; Q4: adjusted OR = 18.22, P = 0.004) in model 2, whereas all models consistently showed no significant association for Q2 group (P > 0.05).

**Fig. 2:**
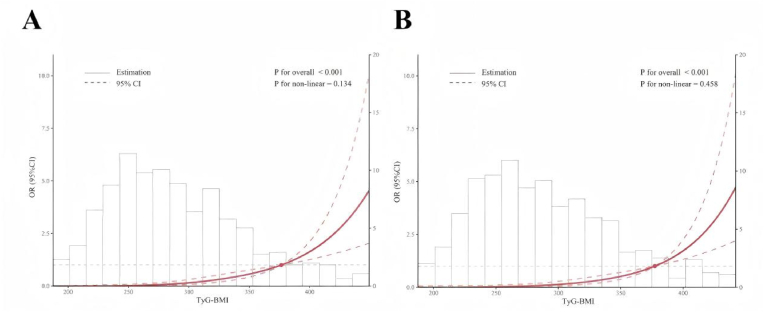
Restricted cubic spline regression analysis of TyG-BMI index with high risk MASH in patients with MASLD before and after sensitivity analysis. A, sensitivity analysis before exclusion of participants receiving insulin treatment. B, sensitivity analysis after exclusion of participants receiving insulin treatment.

RCS was used to explore nonlinear association between the TyG-BMI index and high-risk MASH. The results showed that, either before sensitivity analysis (before exclusion of participants receiving insulin treatment, Fig. 2A) or after sensitivity analysis (after exclusion of participants receiving insulin treatment, Fig. 2B), no nonlinear association was observed between TyG-BMI index and high-risk MASH (before sensitivity analysis: P for non-linear = 0.134; after sensitivity analysis: P for non-linear = 0.458). In a sensitivity analysis employing the more stringent FAST score threshold of ≥0.67 to define high-risk MASH (as shown in Supplementary Table 2), the positive association between TyG-BMI and disease risk was still significant (OR = 1.07, 95 % CI: 1.03–1.11, P = 0.001).

### 3.3 Associations between the TyG-BMI index and CVD in participants with MASLD

After multivariable adjustment, compared with the TyG-BMI index Q1 group, ORs of CVD were 2.85 (95 % CI: 1.13–7.20), 3.25 (95 % CI: 1.26–8.35), and 3.53 (95 % CI: 1.26–9.90) in Q2, Q3, and Q4 groups, respectively (Table 3). After sensitivity analysis (exclusion of participants receiving insulin treatment), the Q2, Q3 and Q4 groups retained significant positive associations (Q2: adjusted OR = 2.66, 95 %CI = 1.03–6.86; Q3: adjusted OR = 3.49, 95 %CI = 1.33–9.11; Q4: adjusted OR = 3.37, 95 %CI = 1.18–9.63) in model 2.

**Table 3:** Associations between TyG-BMI index and CVD (n = 674), NHANES 2017–2020.

	TyG-BMI index			
	Q1	Q2	Q3	Q4
Model 1				
OR (95 %CI) P-value	1.00	2.39(1.05,5.41) 0.037	2.94(1.32,6.54) 0.008	1.96(0.85,4.54) 0.115
Model 2				
OR (95 %)CI P-value	1.00	2.85(1.13,7.20) 0.026	3.25(1.26,8.35) 0.014	3.53(1.26,9.90) 0.017
Sensitivity analysis after exclusion of participants receiving insulin treatment (n = 652)				
Model 1				
OR (95 %CI) P-value	1.00	2.09(0.90,4.83) 0.085	2.93(1.31,6.54) 0.009	1.85(0.79,4.31) 0.155
Model 2				
OR (95 %)CI P-value	1.00	2.66(1.03,6.86) 0.043	3.49(1.33,9.11) 0.011	3.37(1.18,9.63) 0.023

Model 1: Non-adjusted model.

Model 2: Adjusted for:age; gender; race; smoker; PA; hypertension; diabetes; AST; ALT; HbA1c; HDL-C; LDL-C; statin; insulin.

**Fig. 3:**
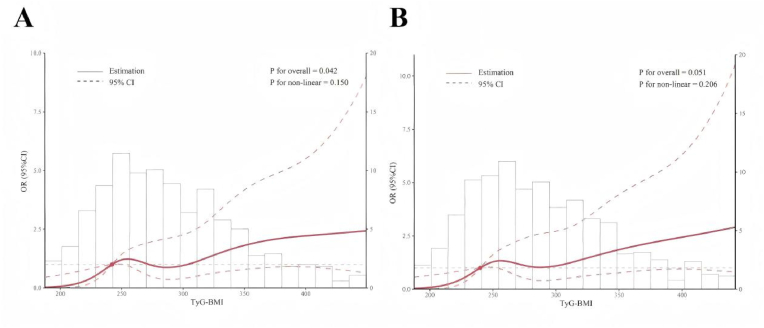
Restricted cubic spline regression analysis of TyG-BMI index with CVD in patients with MASLD before and after sensitivity analysis. A, sensitivity analysis before exclusion of participants receiving insulin treatment. B, sensitivity analysis after exclusion of participants receiving insulin treatment.

As shown in Fig. 3, there was no significant non-linear association between the TyG-BMI index and CVD was observed in patients with MASLD, either before or after the sensitivity analysis (before sensitivity analysis: P for non-linear = 0.150; after sensitivity analysis: P for non-linear = 0.206). A subgroup analysis was conducted to evaluate the associations between TyG-BMI quartiles and specific CVD, including CHF, CHD, heart attack, and stroke. As shown in Supplementary Fig. 1, compared with the Q1, the higher TyG-BMI quartiles were associated with an increased CVD outcomes; however, statistically significant associations were observed only observed for CHF, CHD and heart attack.

### 3.4 Subgroup analysis

Subgroup analyses revealed differential associations between TyG-BMI index and high risk MASH across populations (Table 4). Across the entire cohort, each unit increase in TyG-BMI index corresponded to a 3 % higher risk of developing MASH risk by 3 % (OR = 1.03, 95 %CI:1.01–1.04, P < 0.001). Gender stratified analysis showed a stronger association in men (OR = 1.03, P < 0.001) and no significant association in women (P = 0.117). Notably, smokers exhibited higher risk (OR = 1.07) than did non-smokers (OR = 1.02). The association between the TyG-BMI index and high-risk MASH remained significant regardless of whether PA, diabetes or hypertension was combined(all P < 0.05). Individuals with high-risk MASH showed a significant interaction between gender and hypertension.

**Table 4:** Subgroup analyses of TyG-BMI index with high risk MASH∗.

	Events	OR (95 %CI)	P value	P for interaction
All participants	674	1.03(1.01,1.04)	<0.001	
Gender				<0.001
Man	390	1.03(1.02,1.05)	<0.001	
Woman	284	1.02(1.00,1.04)	0.117	
Smoker				0.082
No	394	1.02(1.01,1.03)	<0.001	
Yes	280	1.07(1.01,1.12)	0.014	
PA				0.664
No	388	1.02(1.01,1.03)	<0.001	
Yes	286	1.06(1.03,1.08)	<0.001	
Hypertension				0.030
No	314	1.05(1.02,1.08)	<0.001	
Yes	360	1.02(1.01,1.04)	0.001	
Diabetes				0.052
No	478	1.03(1.01,1.05)	<0.001	
Yes	196	1.03(1.02,1.05)	<0.001	

Model was adjusted for:age; gender; race; smoker; PA; hypertension; diabetes; AST; ALT; HbA1c; HDL-C; LDL-C; statin; insulin. ∗High risk MASH was dignosised by FAST score ≥0.35.

Subgroup analyses was used to explore associations between TyG-BMI index and CVD (Table 5). In the overall population, each unit increase in TyG-BMI index corresponded to a significant 1 % higher CVD risk (OR = 1.01, 95 %CI:1.00–1.01, P = 0.028). Gender stratification showed that this association was limited to men (OR = 1.01, P = 0.015) and not significant in women (P = 0.359). In addition, there was a significant associations between patients with hypertension (OR = 1.01, P = 0.022) and diabetes (OR = 1.01, P = 0.036), but no significant associations between non-hypertensive patients (P = 0.711) and non-diabetic patients (P = 0.110). For CVD, no significant interaction was observed across all subgroups.

**Table 5:** Subgroup analyses of TyG-BMI index with CVD.

	Events	OR (95 %CI)	P value	P for interaction
All participants	674	1.01(1.00,1.01)	0.028	
Gender				0.105
Man	390	1.01(1.00,1.02)	0.015	
Woman	284	1.00(1.00,1.01)	0.359	
Smoker				0.208
No	394	1.01(1.00,1.02)	0.145	
Yes	280	1.01(1.00,1.01)	0.119	
PA				0.465
No	388	1.01(1.00,1.01)	0.118	
Yes	286	1.01(1.00,1.02)	0.115	
Hypertension				0.095
No	314	1.00(0.99,1.02)	0.711	
Yes	360	1.01(1.00,1.01)	0.022	
Diabetes				0.712
No	478	1.01(1.00,1.01)	0.110	
Yes	196	1.01(1.00,1.02)	0.036	

Model was adjusted for:age; gender; race; smoker; PA; hypertension; diabetes; AST; ALT; HbA1c; HDL-C; LDL-C; statin; insulin.

### 3.5 Machine learning model construction and evaluation

#### 3.5.1 High risk MASH

##### 3.5.1.1 Selection of feature variables

**Fig. 4:**
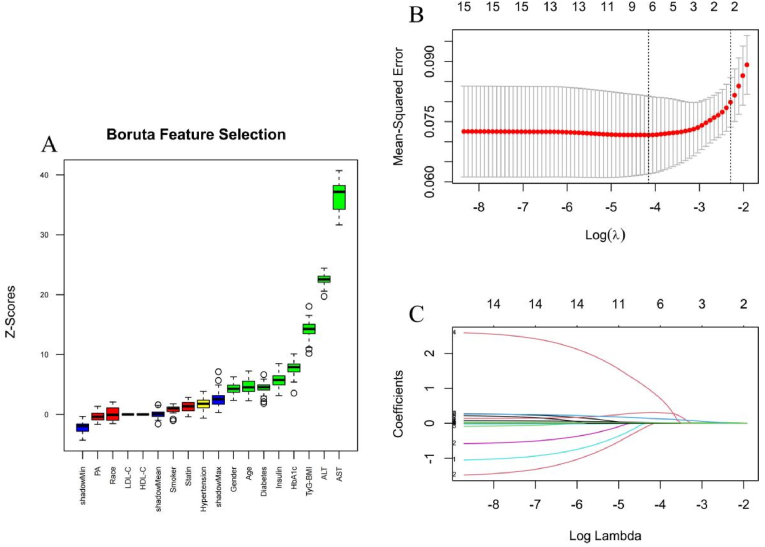
Feature selection for high-risk MASH (FAST ≥0.35) by Boruta algorithm and lasso regression. (A) Feature selection based on the Boruta algorithm. The horizontal axis is the name of each variable, and the vertical axis is the Z value of each variable. The box plot shows the Z value of each variable during model calculation. The green boxes represent important variables, the red boxes represent unimportant variables, and the yellow boxes represent potentially important variables. (B) Lasso regression coefficient paths. (C) Ten-fold cross-validation plots of lasso regression.

For high risk MASH (FAST score ≥0.35), the intersection of features identified by both the Boruta and lasso methods yielded a refined set of six features for the subsequent model construction: age, diabetes, ALT, AST, insulin treatment, and TyG-BMI index (Fig. 4). To further validate the robustness outcome of high-risk MASH (FAST ≥0.67), we performed Boruta algorithm and lasso regression for comprehensive feature selection (Supplementary Fig. 2). Notably, both methods consistently identified the TyG-BMI index as a key variable for prediction, alongside AST. However, Given the limited number of high-risk MASH cases according to the EPV guideline in our study, proceeding with model training would risk overfitting. Therefore, a full machine learning model was not constructed.

##### 3.5.1.2 Model performance evaluation

**Fig. 5:**
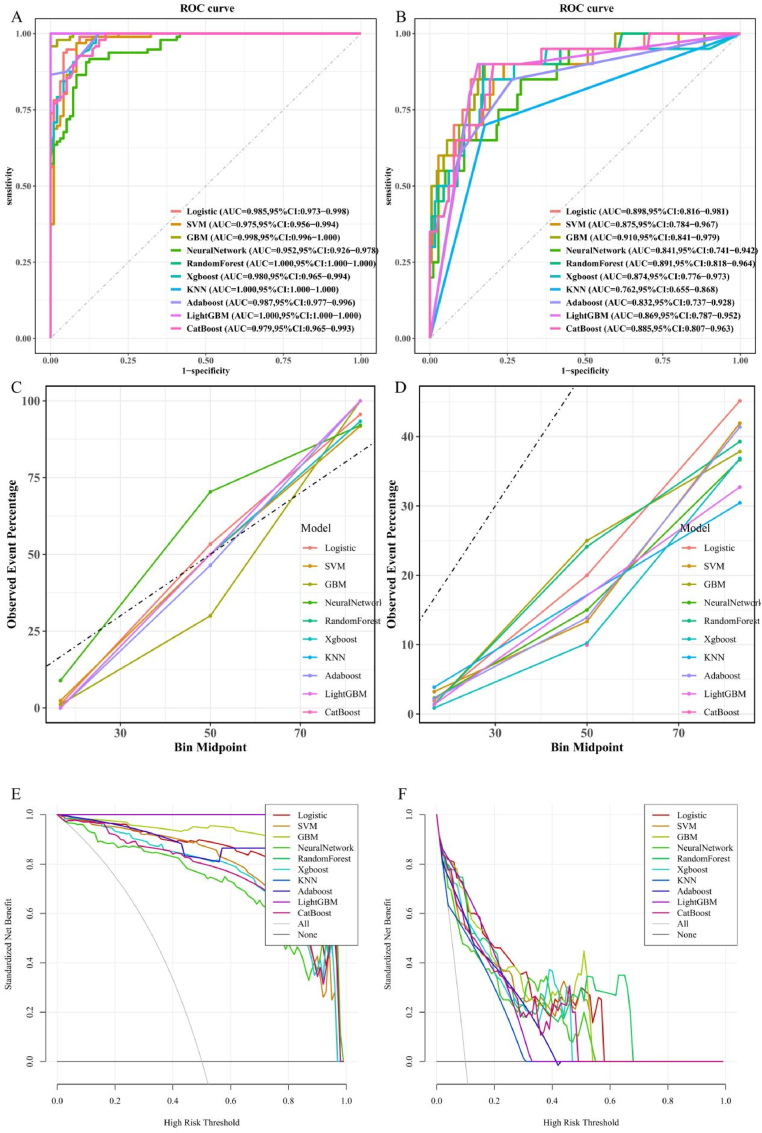
Evaluation the performance of all high-risk MASH (FAST score ≥0.35) machine learning models with ROC, calibration curve, and DCA in the training set (A, C, E) and validation set (B, D, F). AUC, area under curve; CI, confidence interval; DCA, decision curve analysis; ROC, receiver operating characteristic curve; LR, logistic regression; SVM, support vector machine; GBM, gradient boosting machine; NeuralNetwork, neural network; RandomForest, random forest; XGBoost, extreme gradient boosting; KNN, k nearest neighbor; AdaBoost, adaptive boosting; LightGBM, light gradient boosting machine; CatBoost, categorical features gradient boosting.

We constructed 10 models to discriminate high risk MASH (FAST score ≥0.35) in MASLD patients: LR, SVM, GBM, Neural Network, RF, Xgboost, KNN, AdaBoost, Light GBM, and CatBoost. Fig. 5A and B shows the AUC values of these 10 models on the training set and validation set. As shown in Fig. 5B, in validation set, the model with GBM has relatively better fitting performance, with an AUC value of 0.910. Fig. 5C and D presents calibration curves for each model, where GBM model showed the highest consistency between predicted and actual values in validation set. Fig. 5E and F shows the DCA curves of the 10 models, indicating that GBM has the highest clinical net benefit in validation set. In this study, we chose GBM model for further study. The comprehensive performance metrics for all machine learning models in discriminating high-risk MASH are detailed in Supplementary Table 3.

##### 3.5.1.3 Feature importance

**Fig. 6:**
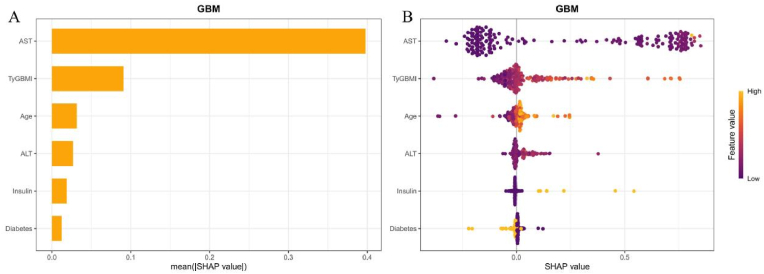
Best high-risk MASH (FAST score ≥0.35) machine learning model explanation by the SHAP method. A, SHAP summary bar chart. This chart evaluates the contribution of each feature to the model by ranking the average SHAP values in descending order. B, SHAP summary dot plot. The probability of high risk MASH increases with the SHAP values of the features. Each point represents the SHAP value of a patient on a particular feature, with yellow indicating higher values and purple indicating lower values. The points are stacked vertically to show density.

As shown in Fig. 6A, the order of Y-axis from top to bottom represents the importance of all features in GBM to high-risk MASH, among which the top three are AST, TyG-BMI index and age. As shown in Fig. 6B, with the increase of TyG-BMI index, its positive contribution to the discriminatory value also increases.

#### 3.5.2 CVD

##### 3.5.2.1 Selection of feature variables

**Fig. 7:**
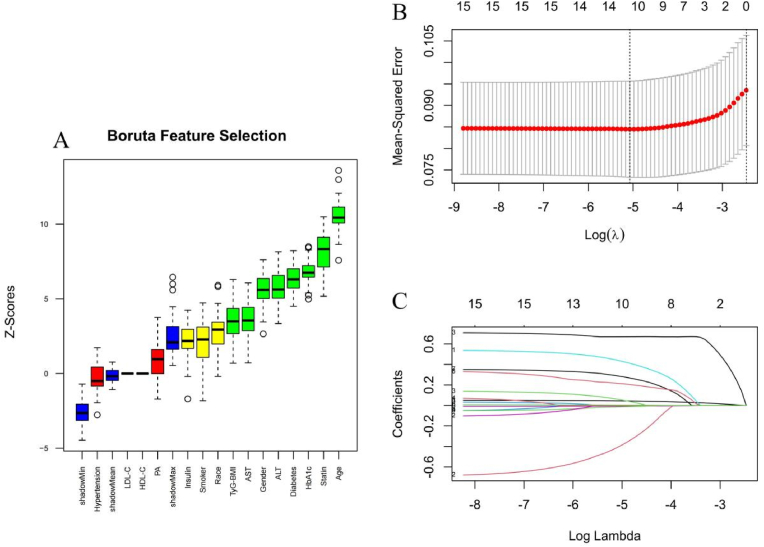
Feature selection for CVD by Boruta algorithm and lasso regression. (A) Feature selection based on the Boruta algorithm. The horizontal axis is the name of each variable, and the vertical axis is the Z value of each variable. The box plot shows the Z value of each variable during model calculation. The green boxes represent important variables, the red boxes represent unimportant variables, and the yellow boxes represent potentially important variables. (B) Lasso regression coefficient paths. (C) Ten-fold cross-validation plots of lasso regression.

For CVD, the intersection of features identified by both the Boruta and lasso methods were: age, gender, statin treatment, diabetes, ALT, and TyG-BMI index(Fig. 7).

##### 3.5.2.2 Model performance evaluation

**Fig. 8:**
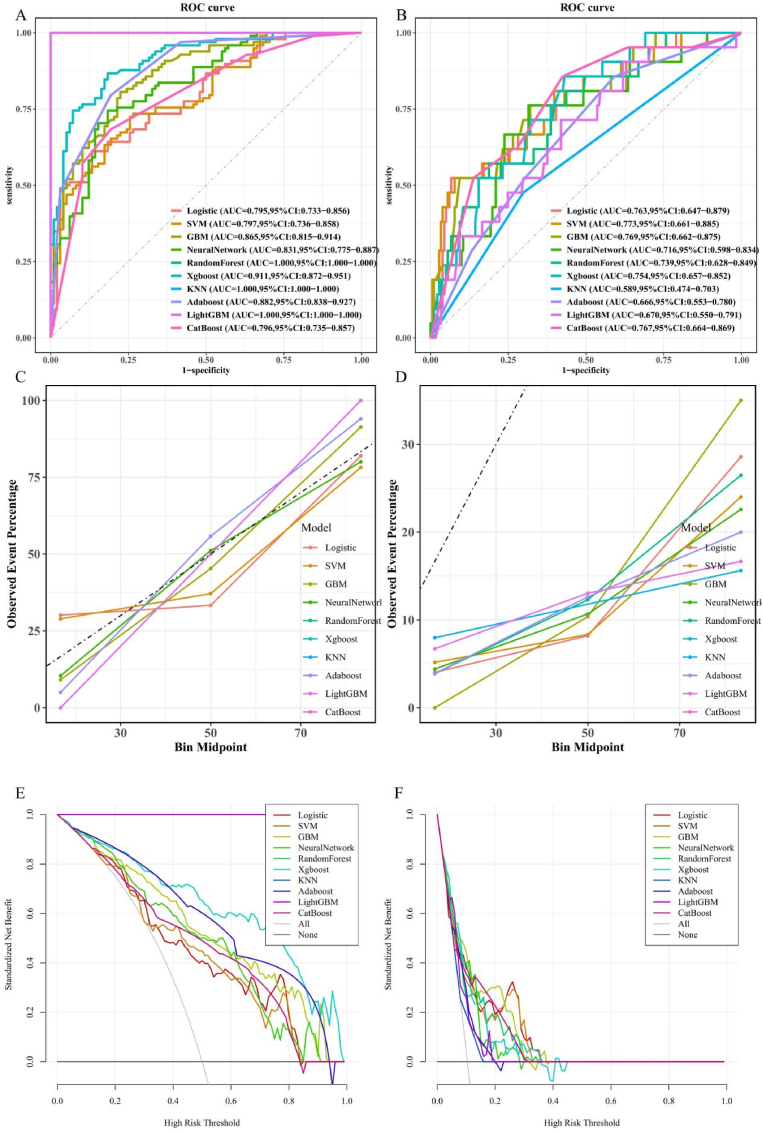
Evaluation the performance of all CVD machine learning models with ROC, calibration curve, and DCA in the training set (A, C) and validation set (B, D). AUC, area under curve; CI, confidence interval; DCA, decision curve analysis; ROC, receiver operating characteristic curve; LR, logistic regression; SVM, support vector machine; GBM, gradient boosting machine; NeuralNetwork, neural network; RandomForest, random forest; XGBoost, extreme gradient boosting; KNN, k nearest neighbor; AdaBoost, adaptive boosting; LightGBM, light gradient boosting machine; CatBoost, categorical features gradient boosting.

Similar to our previous approach, we developed 10 models to discriminate CVD in MASLD patients, as illustrated in Fig. 8. Fig. 8A and B shows the AUC values of these 10 models on the training and validation sets, respectively. The SVM model performed best on the validation set, with an AUC value of 0.773, surpassing the other models. Fig. 8C and D presents calibration curves for each model, where lightGBM and GBM models showed the highest consistency between predicted and actual values in validation set. Fig. 8E and F illustrate the DCA of these 10 models on the training set, where the LR and SVM models demonstrated the greatest clinical net benefit. The model performance metrics for CVD classification are summarized in Supplementary Table 4.

##### 3.5.2.3 Feature importance

**Fig. 9:**
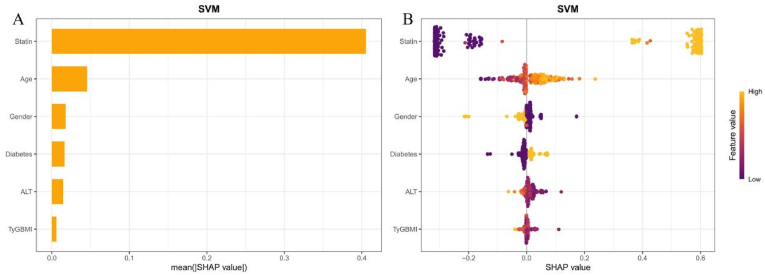
Best CVD machine learning model explanation by the SHAP method. A, SHAP summary bar chart. This chart evaluates the contribution of each feature to the model by ranking the average SHAP values in descending order. B, SHAP summary dot plot. The probability of CVD increases with the increase of SHAP value of the feature. Each point represents the SHAP value of a patient on a particular feature, with yellow indicating higher values and purple indicating lower values. The points are stacked vertically to show density.

The SHAP analysis was used to assess the contribution of features to the model, ranking them by importance. The order of these features was: statin treatment, age, gender, diabetes, ALT and TyG-BMI index (Fig. 9A). The role and direction of these features in the discriminatory model revealed that increase in TyG-BMI index corresponded to greater positive contributions to the model's classification performance (Fig. 9B).

## 4 Discussion

The study included 674 adult participants for analysis and revealed a statistically significant association between elevated TyG-BMI index values and increased risk of both MASH and CVD in patients with MASLD. Patients with higher TyG-BMI index levels had significantly higher rates of high-risk MASH and CVD than did those with lower index levels. After adjusting for multiple covariates, including age, gender, and liver enzymes, the associations between the TyG-BMI index and high risk MASH and CVD remained significant and linear, further confirming the link between higher TyG-BMI index levels and higher rates of high risk MASH and CVD. To ensure the robustness of variable selection, we employed the Boruta algorithm and lasso regression to select features. TyG-BMI index was identified as an important discriminatory factor for both high risk MASH and CVD, consistent with our logistic regression results. These findings suggest that TyG-BMI index has strong ability to discriminate high-risk MASH and CVD among patients with MASLD.

As an emerging technological paradigm, machine learning is widely applied across various medical fields because of its ability to construct robust risk models and enhance discriminatory power [28,29]. Conventional linear modeling approaches are limited by two key constraints: dependence on researcher-driven variable selection and an inability to account for complex nonlinear relationships frequently observed in clinical datasets. In contrast, machine learning models such as XGBoost can develop more robust and generalizable discriminatory models compared to relying on single-model approaches by integrating multiple weaker models with adaptive data processing techniques.

TyG-BMI index integrates the dual pathogenic effects of IR (TyG) and obesity (BMI), offering a more holistic representation of the complex metabolic dysregulation characteristic of MASLD pathogenesis [30]. IR leads to increased lipolysis in adipose tissue, resulting in the release of FFAs in the blood. Liver takes up excess FFAs, promoting the accumulation of triglycerides inside the liver and thereby contributing to the development of hepatic steatosis [6]. The accumulation of FFAs in hepatocytes triggers mitochondrial dysfunction and excessive generation of reactive oxygen species (ROS), which activates inflammatory pathways mediated by cytokines, such as TNF-α and IL-6, leading to hepatocellular injury and MASH(19). Additionally, IR promotes triglyceride synthesis by regulating key enzymes involved in de novo lipogenesis (DNL) in liver. Elevated triglycerides not only reflect abnormal lipid metabolism but also exacerbate hepatic steatosis by promoting the synthesis and release of fatty acids [31]. Moreover, high triglyceride levels are associated with elevated LDL-C and reduced HDL-C, further exacerbating the progression of MASLD. In obese individuals, adipose tissue secretes pro-inflammatory factors, intensifying intrahepatic inflammation and fibrosis [18]. In obese patients, FFA released from visceral fat directly enters the liver via the portal vein, exacerbating lipid toxicity [32]. Our findings regarding the central role of adiposity and dyslipidemia align with recent evidence. Matteis et al. demonstrated that abdominal obesity, rather than BMI-defined obesity, is a potent independent predictor of major adverse cardiovascular events, and that low HDL-c is similarly a key risk factor [33]. Furthermore, a study on the atherogenic index of plasma highlights its strong correlation with metabolic dysfunction and its potential utility as a sensitive biomarker for severe liver steatosis [4].

The TyG index reflects IR and is directly linked to the pathological basis of hepatic steatosis; the inclusion of BMI integrates the synergistic effects of obesity and visceral fat, thereby enhancing diagnostic accuracy. Lim et al. proposed that the TyG-BMI index exhibits superior discriminatory validity compared with the TyG index in predicting IR, which aligns with our findings and suggests that incorporating BMI improves the identification of metabolic abnormalities [34]. Wang et al. found that the TyG-BMI index is more effective than the simple TyG index in predicting NAFLD, as well as severity of hepatic steatosis. The AUC for the TyG-BMI index is significantly higher than that for the TyG index (0.808 vs. 0.720), and a unit increase in the index value indicates increased risk of hepatic steatosis by 1.034 times [35]. Zhang et al. discovered that the TyG index outperforms traditional IR indicators (HOMA-IR) in diagnosing NAFLD, with an AUC of 0.81 [36]. Chang et al. showed that TyG-related indicators (such as TyG-WC) are more effective than a single indicator in identifying metabolic syndrome and NAFLD [37]. Our study provides the first direct evidence, to the best of our knowledge, that the TyG-BMI index can independently distinguish both high-risk MASH and CVD among patients with MASLD, establishing its utility for stratified risk assessment and prognostic evaluation.

Hyperinsulinemia and chronic inflammation, triggered by IR, disrupt vascular endothelial function, promoting the formation of atherosclerotic plaques [38]. Additionally, obesity, particularly the accumulation of visceral fat, releases large amounts of FFAs and pro-inflammatory cytokines, further exacerbating lipid metabolism disorders and vascular inflammation [12]. IR accelerates the ectopic deposition of lipids in liver, leading to the progression of NAFLD, increasing the risk of liver inflammation and fibrosis, and subsequently promoting myocardial oxidative stress and coronary artery disease through the liver-heart axis [39,40]. On the other hand, the lipotoxicity associated with obesity can directly damage myocardial cells and activate the renin-angiotensin system, leading to increased blood pressure and cardiac load [41]. Furthermore, a previous study has shown that hypertriglyceridemia and blood glucose fluctuations can also increase platelet activation and thrombosis, raising the risk of cardiovascular events such as acute coronary syndrome, heart failure, or arrhythmia [42].

Therefore, the TyG-BMI index, as a biomarker, integrates the effects of IR and obesity, reflecting two pathways leading to the progression and worsening of MASLD and development of CVD. The interaction between IR and obesity leads to increased liver inflammation, fibrosis, and cardiovascular risk. In the real world, the TyG-BMI index, as an easily accessible and low-cost indicator, can help physicians quickly identify “high-risk” patients with underlying conditions such as metabolic disorders, diabetes, or hypertension who are at elevated risk of developing MASH or CVD.

The TyG-BMI index exhibits robust and independent discriminative performance, as demonstrated by both traditional statistics and machine learning algorithms, which underscores its potential for clinical application. Given that it is derived from routine, low-cost, and readily available laboratory tests and anthropometric measurements, the TyG-BMI index can be seamlessly integrated into current clinical workflows. Specifically, this index could serve as a valuable tool for risk stratification in primary care and gastroenterology clinics. For instance, physicians could calculate the TyG-BMI index for patients diagnosed with MASLD to identify those at the highest risk of progressing to high-risk MASH or developing CVD. These high-risk individuals could then be prioritized for more intensive lifestyle interventions, closer monitoring, and earlier consideration for pharmacological therapies aimed at mitigating metabolic risk factors. This proactive, risk-based approach could potentially improve patient outcomes by enabling timely and targeted management strategies before the onset of advanced disease complications.

This study has a few limitations. Firstly, the use of a cross-sectional design of the study limits the ability to draw causal inferences in this study. Future multicenter, longitudinal studies are warranted to validate these findings and clarify the causal relationships. Secondly, the diagnosis of high risk MASH is based on the FAST score; future research could consider using liver biopsy to further confirm our findings. In addition, the definition of CVD was defined based on self-reported physician diagnoses, which may be subject to recall bias and misclassification. Future studies incorporating verified medical records are warranted to confirm our findings. Thirdly, our findings revealed a significant association only with CHF, CHD and heart attack, likely due to limited statistical power from the small number of CVD events. Future studies with more CVD cases are needed to validate these associations.Fourthly, despite including many potential confounding factors, the database limitations may have overlooked some factors, such as genetic susceptibility and gut microbiota imbalance. Fifthly, the limited sample size of MASLD patients might affect the statistical power and generalisability of our results, highlighting the need for validation in larger and more diverse populations. The counterintuitive observation of younger age in the highest TyG-BMI quartile, consistent with other cohorts [43], may reflect an aggressive disease subtype or the secular trend of earlier metabolic disease onset. Future longitudinal studies are necessary to elucidate the underlying mechanisms.Furthermore, we could not assess the role of TyG-BMI in lean vs. non-lean MASLD owing to the limited number of cases. Lean MASLD may involve distinct mechanisms, such as genetic risk and oxidative stress, explaining its paradoxically higher mortality despite milder histology [44,45]. Future large-scale studies on lean MASLD are needed for subtype-specific risk stratification. Finally, the machine learning-based discriminatory models developed to discriminate high-risk MASH and CVD require external validation, and interventions for high risk individuals need to be developed.

## 5 Conclusion

The TyG-BMI index significantly impacts the likelihood of high risk MASH and CVD in patients with MASLD. Early identification and monitoring of this index may facilitate the assessment of MASLD progression and inform future therapeutic approaches. Further research, particularly prospective studies involving external validation cohorts, is warranted to validate and extend these findings.

## CRediT authorship contribution statement

**Xiaoxuan Tang:** Writing – review & editing, Writing – original draft, Visualization, Validation, Supervision, Software, Resources, Project administration, Methodology, Investigation, Formal analysis, Data curation, Conceptualization. **Fenglan Wang:** Formal analysis. **Yiran Liu:** Writing – review & editing, Data curation. **Yujia Gao:** Writing – review & editing, Writing – original draft, Data curation. **Mengxi Li:** Data curation. **Chong Liu:** Data curation. **Duanming Zhuang:** Methodology, Formal analysis. **Bin Zhang:** Visualization, Validation, Project administration, Funding acquisition, Formal analysis.

## Ethics approval

This ethics review board of the National Center for Health Statistics approved all NHANES protocols.

## Consent for publication

Not applicable.

## Funding

Funded by the Nanjing Health Science and Technology Development Foundation (YKK24073), 10.13039/501100004608Natural Science Foundation of Jiangsu Province, China (BK20191119) and 10.13039/501100013059Jiangsu Provincial Medical Youth Talent (QNRC2016031).

## Conflict of interests

The authors declare no competing interests.

## Data Availability

The data used in this study are publicly available in the NHANES database (www.cdc.gov/nchs/nhanes).
