# Supplementary material for: Association between triglyceride glucose-body mass index and MASH, cardiovascular disease in MASLD patients: a cross-sectional study and machine learning analysis
Source: Metabol Open. 2025 Nov 19;28:100423. doi: 10.1016/j.metop.2025.100423 (PMC12670593; doi:10.1016/j.metop.2025.100423)
Supplement: Multimedia component 1 [file mmc1.docx]

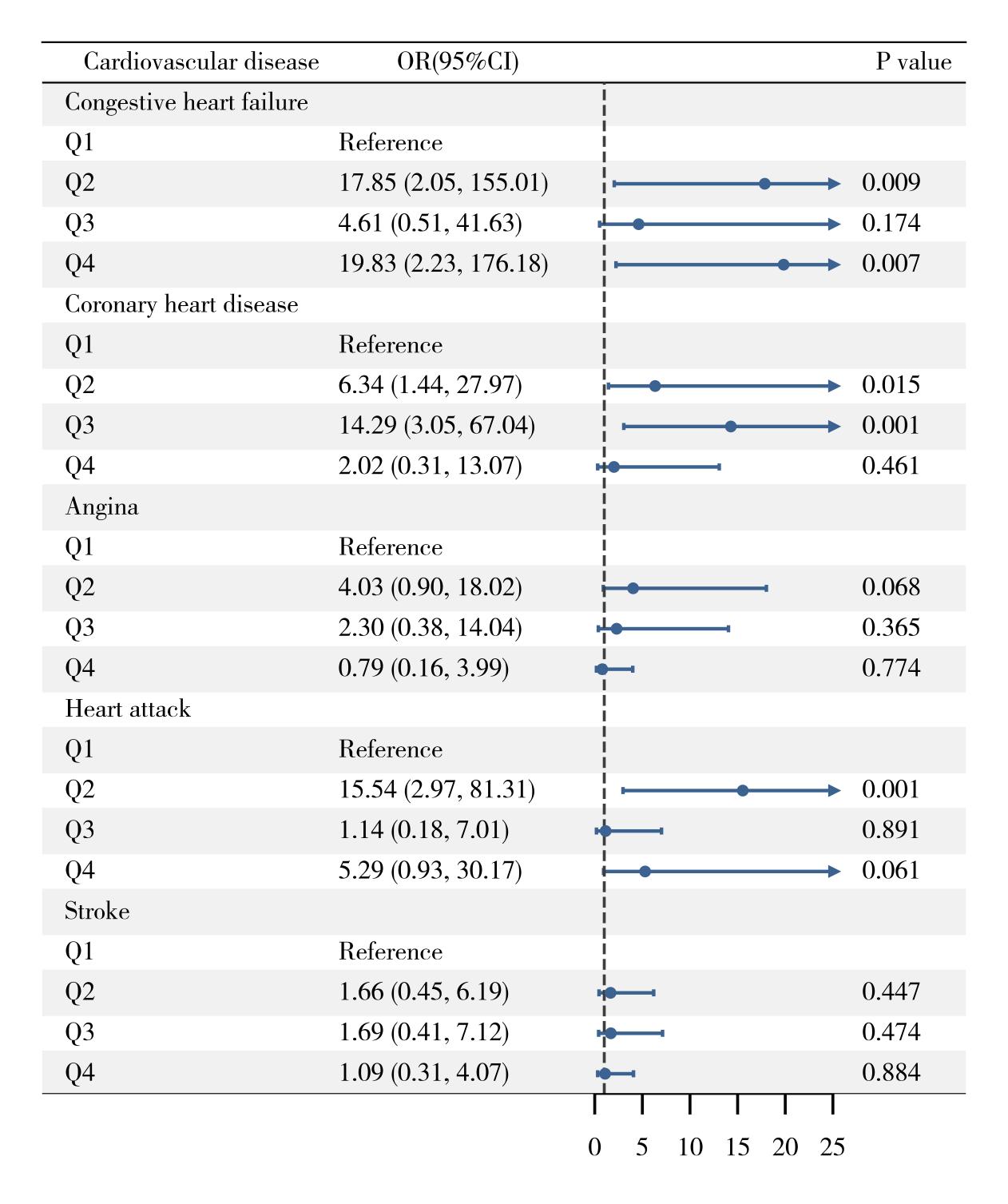


**Supplementary Figure 1**: Forest Plot for the Associations between TyG-BMI and Various Cardiovascular Disease Outcomes in Patients with MASLD

**
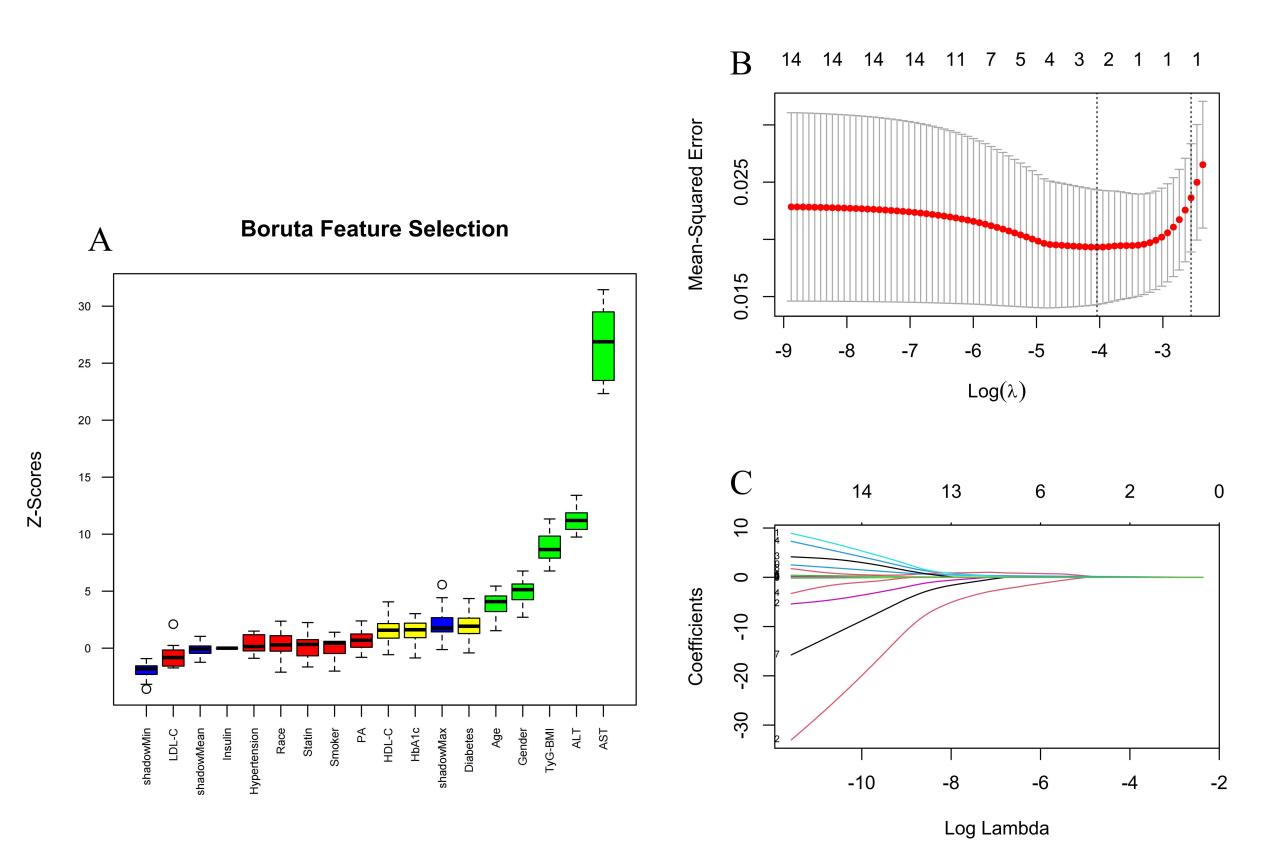
**

**Supplementary Figure 2**: Feature selection for high risk MASH (FAST ≥ 0.67) by Boruta algorithm and lasso regression. (A) Feature selection based on the Boruta algorithm. The horizontal axis is the name of each variable, and the vertical axis is the Z value of each variable. The box plot shows the Z value of each variable during model calculation. The green boxes represent important variables, the red boxes represent unimportant variables, and the yellow boxes represent potentially important variables. (B) Lasso regression coefficient paths. (C) Ten-fold cross-validation plots of lasso regression.

**
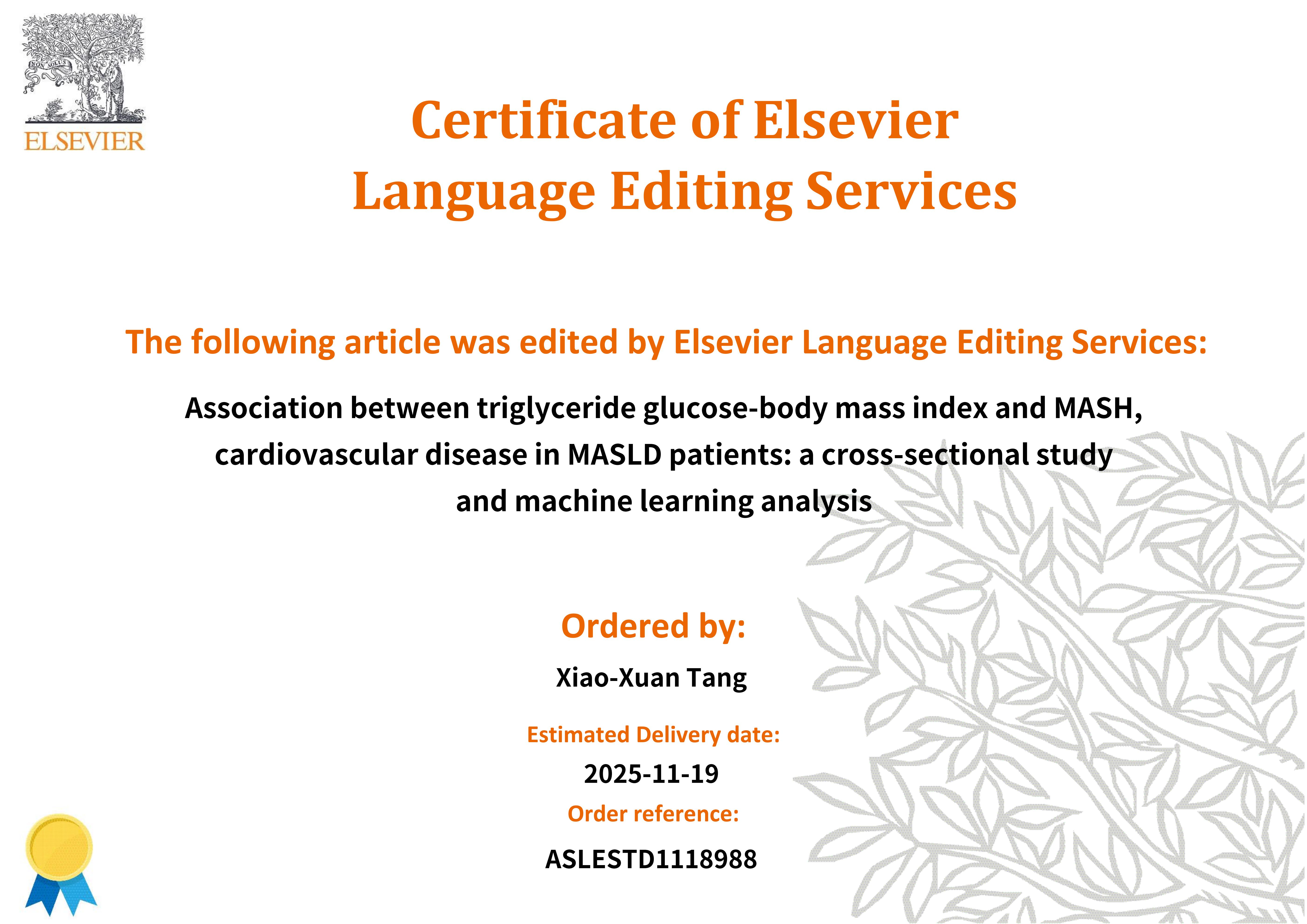
**

**Supplementary figure 3**: Certificate of Elsevier Language Editing Services**.**

**Supplementary table 1**: Baseline characteristics of patients grouped according to FAST score.

| Characters | FAST score | | | P value |
| --- | --- | --- | --- | --- |
|  | <0.35 (n=606) | 0.35-0.66 (n=51) | ****≥****0.67 (n=17) |  |
| Demographic Characteristics |  |  |  |  |
| Age(years) | 54.733 [39.000, 65.000] | 42.000 [34.078, 61.000] | 49.280 [42.543, 55.805] | 0.423 |
| Gender,n(%) |  |  |  | 0.001 |
| Man | 339 (57.01%) | 37 (77.00%) | 14 ( 92.28%) |  |
| Woman | 267 (42.99%) | 14 (23.00%) | 3 ( 7.72%) |  |
| Race,n(%) |  |  |  | 0.130 |
| Mexican American | 88 ( 9.66%) | 9 (12.14%) | 4 ( 10.13%) |  |
| Other Hispanic | 58 ( 5.78%) | 10 (20.57%) | 0 ( 0.00%) |  |
| Non-Hispanic White | 239 (69.09%) | 15 (51.83%) | 7 ( 77.44%) |  |
| Non-Hispanic Black | 126 ( 8.20%) | 10 ( 7.92%) | 2 ( 3.71%) |  |
| Other Race | 95 ( 7.27%) | 7 ( 7.54%) | 4 ( 8.72%) |  |
| Lifestyle Factors |  |  |  |  |
| Smoker,n(%) |  |  |  | 0.730 |
| No | 350 (59.91%) | 34 (63.90%) | 10 ( 48.19%) |  |
| Yes | 256 (40.09%) | 17 (36.10%) | 7 ( 51.81%) |  |
| PA,n(%) |  |  |  | 0.144 |
| No | 350 (59.03%) | 32 (51.77%) | 6 ( 28.90%) |  |
| Yes | 256 (40.97%) | 19 (48.23%) | 11 ( 71.10%) |  |
| Disease Status |  |  |  |  |
| Hypertension,n(%) |  |  |  | 0.048 |
| No | 291 (52.03%) | 16 (24.82%) | 7 ( 62.88%) |  |
| Yes | 315 (47.97%) | 35 (75.18%) | 10 ( 37.12%) |  |
| Diabetes,n(%) |  |  |  | 0.123 |
| No | 443 (81.35%) | 26 (66.57%) | 9 ( 68.22%) |  |
| Yes | 163 (18.65%) | 25 (33.43%) | 8 ( 31.78%) |  |
| Laboratory Parameters |  |  |  |  |
| AST(U/L) | 20.000 [17.000, 23.000] | 34.765 [27.000, 43.000] | 39.184 [21.000, 57.853] | <0.001 |
| ALT(U/L) | 21.000 [16.000, 28.000] | 53.000 [36.000, 63.715] | 49.830 [30.000, 63.144] | <0.001 |
| HbA1c(%) | 5.600 [5.300, 5.900] | 5.615 [5.400, 6.300] | 5.844 [5.333, 6.300] | 0.274 |
| HDL-C(mg/dL) | 48.000 [41.000, 58.000] | 39.079 [34.537, 45.000] | 41.343 [37.000, 43.169] | 0.002 |
| LDL-C(mg/dL) | 114.000 [88.000, 135.038] | 113.548 [93.912, 131.000] | 119.000 [92.637, 119.026] | 0.983 |
| Treatment |  |  |  |  |
| Statin,n(%) |  |  |  | 0.439 |
| No | 432 (71.01%) | 35 (80.53%) | 13 ( 77.78%) |  |
| Yes | 174 (28.99%) | 16 (19.47%) | 4 ( 22.22%) |  |
| Insulin,n(%) |  |  |  | 0.197 |
| No | 589 (98.42%) | 46 (93.37%) | 17 (100.00%) |  |
| Yes | 17 ( 1.58%) | 5 ( 6.63%) | 0 ( 0.00%) |  |
| TyG-BMI index | 275.928 [243.783, 319.911] | 318.817 [292.194, 364.618] | 370.723 [298.063, 442.430] | <0.001 |

Values are weighted.Bold indicates P value<0.05. MASLD, metabolic dysfunctionassociated steatotic liver disease; PA, physical activity – meeting MET ( ≥ 600 MET-minutes/week, equivalent to 150 min/week of moderate-intensity or 75 min/week of vigorous-intensity physical activity); MET: metabolic equivalent minutes of moderate to vigorous physical activity per week; AST, aspartate aminotransferase; ALT, alanine aminotransferase; HbA1c, glycated hemoglobin; HDL-C, high density lipoprotein cholesterol; LDL-C, low density lipoprotein cholesterol; FAST, FibroScan-AST.

**Supplementary table 2**: Associations between TyG-BMI index and high risk MASH, NHANES 2017–2020.

|  | Crude model | | Adjusted model | |
| --- | --- | --- | --- | --- |
|  | OR (95%CI) | P value | OR (95%CI) | P value |
| High risk MASH was diagnosised by FAST score ≥ 0.35 | | | | |
| TyG-BMI index | 1.01 (1.01,1.02) | <0.001 | 1.02 (1.01,1.03) | 0.001 |
| High risk MASH was diagnosised by FAST score ≥ 0.67 | | | | |
| TyG-BMI index | 1.02 (1.01,1.03) | 0.004 | 1.07 (1.03, 1.11) | 0.001 |

Crude model : Non-adjusted model;

Adjusted model: Adjusted for gender, hypertension, AST, ALT and HDL-C.

**Supplementary table 3**: Performance of machine learning algorithms for high risk MASH (FAST score ≥ 0.35).

| **Training set** | | | | | | |
| --- | --- | --- | --- | --- | --- | --- |
| Model | Brier score | Threshold | Accuracy | Sensitivity | Specificity | Precision |
| Logistic | 0.043 | 0.377 | 0.948 | 0.990 | 0.906 | 0.913 |
| SVM | 0.054 | 0.546 | 0.943 | 0.969 | 0.917 | 0.921 |
| GBM | 0.023 | 0.668 | 0.979 | 0.958 | 1.000 | 1.000 |
| Neural Network | 0.092 | 0.365 | 0.896 | 0.917 | 0.875 | 0.880 |
| Random Forest | 0.000 | 0.500 | 1.000 | 1.000 | 1.000 | 1.000 |
| Xgboost | 0.098 | 0.359 | 0.922 | 1.000 | 0.844 | 0.865 |
| KNN | 0.000 | 0.500 | 1.000 | 1.000 | 1.000 | 1.000 |
| Adaboost | 0.037 | 0.759 | 0.932 | 0.865 | 1.000 | 1.000 |
| LightGBM | 0.002 | 0.500 | 1.000 | 1.000 | 1.000 | 1.000 |
| CatBoost | 0.250 | 0.616 | 0.922 | 0.927 | 0.917 | 0.918 |
| **Validation set** | | | | | | |
| Model | Brier score | Threshold | Accuracy | Sensitivity | Specificity | Precision |
| Logistic | 0.100 | 0.304 | 0.846 | 0.900 | 0.840 | 0.383 |
| SVM | 0.114 | 0.203 | 0.776 | 0.900 | 0.762 | 0.295 |
| GBM | 0.118 | 0.405 | 0.831 | 0.900 | 0.823 | 0.360 |
| Neural Network | 0.135 | 0.328 | 0.721 | 0.850 | 0.707 | 0.243 |
| Random Forest | 0.105 | 0.415 | 0.836 | 0.900 | 0.829 | 0.367 |
| Xgboost | 0.154 | 0.465 | 0.831 | 0.850 | 0.829 | 0.354 |
| KNN | 0.189 | 0.500 | 0.811 | 0.700 | 0.823 | 0.304 |
| Adaboost | 0.144 | 0.241 | 0.746 | 0.850 | 0.735 | 0.262 |
| LightGBM | 0.180 | 0.958 | 0.851 | 0.900 | 0.845 | 0.391 |
| CatBoost | 0.349 | 0.615 | 0.806 | 0.900 | 0.796 | 0.327 |

**Supplementary table 4**: Performance of machine learning algorithms for CVD.

| **Training set** | | | | | | |
| --- | --- | --- | --- | --- | --- | --- |
| Model | Brier score | Threshold | Accuracy | Sensitivity | Specificity | Precision |
| Logistic | 0.187 | 0.659 | 0.740 | 0.612 | 0.867 | 0.822 |
| SVM | 0.188 | 0.389 | 0.735 | 0.735 | 0.735 | 0.735 |
| GBM | 0.177 | 0.463 | 0.791 | 0.806 | 0.776 | 0.782 |
| Neural Network | 0.169 | 0.552 | 0.781 | 0.745 | 0.816 | 0.802 |
| Random Forest | 0.000 | 0.500 | 1.000 | 1.000 | 1.000 | 1.000 |
| Xgboost | 0.237 | 0.500 | 0.842 | 0.867 | 0.816 | 0.825 |
| KNN | 0.000 | 0.500 | 1.000 | 1.000 | 1.000 | 1.000 |
| Adaboost | 0.136 | 0.500 | 0.801 | 0.796 | 0.806 | 0.804 |
| LightGBM | 0.000 | 0.503 | 1.000 | 1.000 | 1.000 | 1.000 |
| CatBoost | 0.264 | 0.622 | 0.745 | 0.684 | 0.806 | 0.779 |
| **Validation set** | | | | | | |
| Model | Brier score | Threshold | Accuracy | Sensitivity | Specificity | Precision |
| Logistic | 0.200 | 0.769 | 0.891 | 0.524 | 0.934 | 0.478 |
| SVM | 0.198 | 0.770 | 0.881 | 0.524 | 0.923 | 0.440 |
| GBM | 0.205 | 0.505 | 0.688 | 0.762 | 0.680 | 0.216 |
| Neural Network | 0.214 | 0.516 | 0.693 | 0.762 | 0.685 | 0.219 |
| Random Forest | 0.199 | 0.599 | 0.787 | 0.571 | 0.812 | 0.261 |
| Xgboost | 0.243 | 0.494 | 0.599 | 0.857 | 0.569 | 0.188 |
| KNN | 0.322 | 0.500 | 0.678 | 0.476 | 0.702 | 0.156 |
| Adaboost | 0.248 | 0.223 | 0.455 | 0.857 | 0.409 | 0.144 |
| LightGBM | 0.297 | 0.190 | 0.594 | 0.714 | 0.580 | 0.165 |
| CatBoost | 0.361 | 0.622 | 0.604 | 0.857 | 0.575 | 0.189 |

**Supplementary table 5**: Baseline characteristics of participants in training set and validation set for high risk MASH (FAST score ≥ 0.35) machine learning model.

|  | Training set | Validation set | P value |
| --- | --- | --- | --- |
| High risk MASH, n(%) |  |  | 0.938 |
| No | 425 (89.85%) | 181 (90.05%) |  |
| Yes | 48 (10.15%) | 20 (9.95%) |  |
| Age(years) | 52.195±16.580 | 54.239±15.087 | 0.133 |
| Diabetes, n (%) |  |  | 0.933 |
| No | 335 (70.82%) | 143 (71.14%) |  |
| Yes | 138 (29.18%) | 58 (28.86%) |  |
| ALT(U/L) | 21.000 (16.000, 30.000) | 22.000 (16.000, 30.000) | 0.632 |
| AST(U/L) | 20.000 (16.000, 24.000) | 20.000 (17.000, 25.000) | 0.391 |
| Insulin,n(%) |  |  | 0.835 |
| No | 458 (96.83%) | 194 (96.52%) |  |
| Yes | 15 (3.17%) | 7 (3.48%) |  |
| TyG-BMI index | 289.455±59.816 | 291.778±64.102 | 0.652 |

**Supplementary table 6**: Baseline characteristics of participants in training set and validation set for CVD machine learning model.

|  | Training set | Validation set | P value |
| --- | --- | --- | --- |
| CVD, n(%) |  |  | 0.995 |
| No | 423 (89.62%) | 181 (89.60%) |  |
| Yes | 49 (10.38%) | 21 (10.40%) |  |
| Age(years) | 52.367±16.609 | 53.827±15.068 | 0.283 |
| Gender,n(%) |  |  | 0.508 |
| Man | 277 (58.69%) | 113 (55.94%) |  |
| Woman | 195 (41.31%) | 89 (44.06%) |  |
| Statin,n(%) |  |  | 0.145 |
| No | 344 (72.88%) | 136 (67.33%) |  |
| Yes | 128 (27.12%) | 66 (32.67%) |  |
| Diabetes, n (%) |  |  | 0.126 |
| No | 343 (72.67%) | 135 (66.83%) |  |
| Yes | 129 (27.33%) | 67 (33.17%) |  |
| ALT(U/L) | 21.000 (15.000, 30.000) | 23.000 (17.000, 30.750) | 0.033 |
| TyG-BMI index | 289.652±62.818 | 291.304±56.971 | 0.748 |

**Supplementary table 7**:Data dictionary for the NHANES study.

| **Variables** | **Code** | **Description** |
| --- | --- | --- |
| Age | RIDAGEYR | Age in years at screening |
| Gender | RIAGENDR | Gender |
| Race | RIDRETH1 | Race/Hispanic origin |
| Smoker | SMQ020 | Smoked at least 100 cigarettes in life |
| PA | PAQ605 | Vigorous work activity |
|  | PAQ610 | Number of days vigorous work |
|  | PAD615 | Minutes vigorous-intensity work |
|  | PAQ620 | Moderate work activity |
|  | PAQ625 | Number of days moderate work |
|  | PAD630 | Minutes moderate-intensity work |
| Hypertension | BPXOSY1 | Systolic - 1st oscillometric reading |
|  | BPXODI1 | Diastolic - 1st oscillometric reading |
|  | BPXOSY2 | Systolic - 2nd oscillometric reading |
|  | BPXODI2 | Diastolic - 2nd oscillometric reading |
|  | BPXOSY3 | Systolic - 3rd oscillometric reading |
|  | BPXODI3 | Diastolic - 3rd oscillometric reading |
|  | BPQ020 | Ever told you had high blood pressure |
|  | BPQ040A | Taking prescription for hypertension |
| Diabetes | LBXGLU | Fasting Glucose (mg/dL) |
|  | LBXGH | Glycohemoglobin (%) |
|  | DIQ010 | Doctor told you have diabetes |
|  | DIQ050 | Taking insulin now |
|  | DIQ070 | Take diabetic pills to lower blood sugar |
|  | RXDDRGID for insulin | d00262, d04369, d04370, d04371. d04372, d04373, d04374, d04510, d04538, d04697, d04839, d05278, d05436, d05765, d08054 |
| AST | LBXSASSI | Aspartate Aminotransferase (AST) (IU/L) |
| ALT | LBXSATSI | Alanine Aminotransferase (ALT) (IU/L) |
| HbA1c | LBXGH | Glycohemoglobin (%) |
| HDL-C | LBDHDD | Direct HDL-Cholesterol (mg/dL) |
| LDL-C | LBDLDL | LDL-Cholesterol, Friedewald (mg/dL) |
| Statin | RXDDRGID |  |
|  | c00173 | HMG-COA REDUCTASE INHIBITORS - UNSPECIFIED |
|  | d00280 | LOVASTATIN |
|  | d00348 | PRAVASTATIN |
|  | d00746 | SIMVASTATIN |
|  | d03183 | FLUVASTATIN |
|  | d04105 | ATORVASTATIN |
|  | d04140 | CERIVASTATIN |
|  | d04851 | ROSUVASTATIN |
|  | d07637 | PITAVASTATIN |
| Insulin | RXDDRGID |  |
|  | d00262 | INSULIN |
|  | d04369 | INSULIN REGULAR |
|  | d04370 | INSULIN ISOPHANE (NPH) |
|  | d04371 | INSULIN ZINC |
|  | d04372 | INSULIN ZINC EXTENDED |
|  | d04373 | INSULIN LISPRO |
|  | d04374 | INSULIN ISOPHANE; INSULIN REGULAR |
|  | d04510 | INSULIN LISPRO; INSULIN LISPRO PROTAMINE |
|  | d04538 | INSULIN GLARGINE |
|  | d04697 | INSULIN ASPART |
|  | d04839 | INSULIN ASPART; INSULIN ASPART PROTAMINE |
|  | d05278 | INSULIN GLULISINE |
|  | d05436 | INSULIN DETEMIR |
|  | d05765 | INSULIN INHALATION RAPID ACTING |
|  | d08054 | INSULIN DEGLUDEC |
|  | DIQ050 | Taking insulin now |
| LSM | LUXSMED | Median stiffness (E), kilopascals (kPa) |
| CAP | LUXCAPM | Median CAP, decibels per meter (dB/m) |
| High-risk MASH | LUXSMED | Median stiffness (E), kilopascals (kPa) |
|  | LUXCAPM | Median CAP, decibels per meter (dB/m) |
|  | LBXSASSI | Aspartate Aminotransferase (AST) (IU/L) |
| CVD | MCQ160b | Ever told had congestive heart failure |
|  | MCQ160c | Ever told you had coronary heart disease |
|  | MCQ160d | Ever told you had angina/angina pectoris |
|  | MCQ160e | Ever told you had heart attack |
|  | MCQ160f | Ever told you had a stroke |
| TyG-BMI index | LBXTR | Triglyceride (mg/dL) |
|  | LBXGLU | Fasting Glucose (mg/dL) |
|  | BMXBMI | Body Mass Index (kg/m**2) |
